# Tricuspid Regurgitation and TAVR: Outcomes, Risk Factors and Biomarkers

**DOI:** 10.3390/jcm13051474

**Published:** 2024-03-04

**Authors:** Thomas Puehler, Nina Sophie Pommert, Sandra Freitag-Wolf, Hatim Seoudy, Markus Ernst, Assad Haneya, Janarthanan Sathananthan, Stephanie L. Sellers, David Meier, Jan Schöttler, Oliver J. Müller, Mona Salehi Ravesh, Mohammed Saad, Derk Frank, Georg Lutter

**Affiliations:** 1 Department of Cardiac and Vascular Surgery, University Medical Center Schleswig-Holstein, Campus Kiel, Arnold-Heller Strasse 3, D-24105 Kiel, Germany; ninasophie.pommert@uksh.de (N.S.P.); markus.ernst@uksh.de (M.E.); jan.schoettler@uksh.de (J.S.); georg.lutter@uksh.de (G.L.); 2 DZHK (German Centre for Cardiovascular Research), Partner Site Hamburg/Kiel/Lübeck, 23562 Lübeck, Germany; oliver.mueller@uksh.de (O.J.M.); derk.frank@uksh.de (D.F.); 3 Institute of Medical Informatics and Statistics, University Medical Center Schleswig-Holstein, Campus Kiel, D-24105 Kiel, Germany; freitag@medinfo.uni-kiel.de; 4 Department of Internal Medicine III (Cardiology, Angiology, and Critical Care), University Medical Center Schleswig-Holstein, Campus Kiel, D-24105 Kiel, Germany; hatim.seoudy@uksh.de (H.S.); mohammed.saad@uksh.de (M.S.); 5 Center for Heart Valve Innovation & Cardiovascular Translational Laboratory, St Paul’s Hospital, University of British Columbia, Vancouver, BC V5Z 1M9, Canada; jsathananthan@providencehealth.bc.ca (J.S.); ssellers@providencehealth.bc.ca (S.L.S.); david.meier1291@gmail.com (D.M.); 6 Department of Radiology and Neuroradiology, University Medical Center Schleswig-Holstein, Campus Kiel, D-24105 Kiel, Germany; mona.salehiravesh@uksh.de

**Keywords:** tricuspid regurgitation, tricuspid insufficiency, tricuspid valve, transcatheter aortic valve replacement, implantation, TAVR, aortic stenosis, TR grading, high sensitivity troponin T, N-terminal pro-B-type natriuretic peptide

## Abstract

**Background**. The significance of concomitant tricuspid regurgitation (TR) in the context of transcatheter aortic valve replacement (TAVR) remains unclear. This study aimed to analyze the severity of TR before and after TAVR with regard to short- and long-term survival and to analyze the influencing factors. **Methods**. In our retrospective analysis, TR before and after TAVR was examined and patients were classified into groups accordingly. Special attention was paid to patients with post-interventional changes in TR. Mortality after TAVR was considered the primary endpoint of the analysis and major complications according to the Valve Academic Research Consortium 3 (VARC3) were compared. Moreover, biomarkers and risk factors for worsening or improvement of TR through TAVR were analyzed. **Results**. Among 775 patients who underwent TAVR in our center between January 2009 and December 2019, 686 patients (89%) featured low- and 89 patients (11%) high-grade TR. High-grade pre-TAVR TR was associated with worse short- (30-day), mid- (2-year) and long-term survival up to 8 years. Even though in nearly half of the patients with high-grade TR the regurgitation improved within seven days after TAVR (n = 42/89), this did not result in a survival benefit for this subgroup. On the other hand, a worsening of low-grade TR was seen in more than 10% of the patients (n = 73/686), which was also associated with a worse prognosis. Predictors of worsening of TR after TAVR were adipositas, impaired right ventricular function and the presence of mild TR. Age, atrial fibrillation, COPD, impaired renal function and elevated cardiac biomarkers were risk factors for mortality after TAVR independent from the grade of TR. **Conclusions**. Not only pre-interventional, but also post-TAVR high-grade TR is associated with a worse prognosis after TAVR. TAVR can change concomitant tricuspid regurgitation, but improvement does not have any impact on short- and long-term survival. Worsening of TR after TAVR is possible and impairs the prognosis.

## 1. Introduction

Little is known about the impact of multi-valvular disease on patients’ long-term outcomes after TAVR. In particular, the impact of concomitant tricuspid valve regurgitation (TR), which is present in a greater than moderate severity in up to 20% of patients with aortic stenosis (AS), remains unclear [1,2,3,4,5]. Previous studies have already demonstrated that TR is associated with poor clinical outcomes after TAVR. Shamekhi et al. reported a significantly higher one-year mortality after TAVR in patients with more than mild TR, with an even worse prognosis, where TR was massive or torrential [6]. 

The pathology of TR in cases of AS is often secondary in over 90% of patients due to secondary pulmonary hypertension and right heart strain. Therefore, TR improvement after TAVR could be imagined and has already been described [6,7]. However, data on long-term outcome after improvement or worsening of TR, as well as influencing factors in the context of TAVR, are still incomplete.

## 2. Methods

### 2.1. Study Design

This is a single-center retrospective observational study of 775 patients with aortic stenosis undergoing TAVR at the University Medical Center Schleswig-Holstein, Campus Kiel, Germany, between January 2009 and January 2019. Data were sampled prospectively in the institutional database. The follow-up was done by phone calls to the patient or a call to the supervising family doctor and the registration office. None of the patients in our study were lost to follow-up.

The underlying study was approved by the Local Ethics Committee (AZ D529/16, 13 August 2016) and was conducted in accordance with the Ethical Guidelines of the World Health Organization. The study was registered at the German Center of Clinical Studies (DRKS00022551). Patients’ informed consent to take part in clinical studies conducted at our center was obtained. 

### 2.2. Patient Cohort and Evaluation of TR

Aortic stenosis was diagnosed and managed according to the current ESC guidelines and all patients underwent evaluation before the procedure through a multidisciplinary structural Heart Team. The operative risk for each patient was calculated based on two different scores (EuroScore II and STS Score) [1]. 

Patients undergoing TAVR in our center during the aforementioned period were included in this study. Therefore, we analyzed patients with stenosis of the native aortic valve as well as patients with degenerated bioprosthetic valves. Patients with combined aortic stenosis and insufficiency were also included. Exclusion criteria were the following: waiver of content, incomplete medical records or incomplete echocardiographic examination. This led to the exclusion of 20 of 795 patients (2.5% exclusion, 2.1% incomplete echocardiographic records). A complete echocardiographic examination was defined as having an echocardiogram performed preoperatively and postoperatively at discharge. 

TR severity was determined as recommended by the ESC guidelines and the American Society of Echocardiography using a five-tier reporting model: none (0), trace (1), mild (2), moderate (3) and severe (4) TR [1,8]. Jet area, vena contracta width and regurgitation orifice area measured by the PISA method were considered. Furthermore, right atrial (RA) and right ventricular (RV) sizes, as well as right ventricular function measured by tricuspid annular plane systolic excursion (TAPSE), were assessed by transthoracic echocardiography. With regard to clinical practice and potential treatment, patients were divided into two groups according to their TR grade pre-TAVR: the first group summarized patients with no, trace and mild TR (low-grade TR, TR ≤ 2), and the second group consisted of patients with moderate and severe TR (high-grade TR, TR > 2). 

### 2.3. Endpoints

The primary endpoint for the analysis was mortality after TAVR (30-day, 2-year, and long-term mortality for up to 8 years) as a function of pre-interventional TR. Moreover, major complications in the different groups were assessed according to the Valve Academic Research Consortium 3 (VARC3-criteria): myocardial infarction, stroke, bleeding, acute kidney injury, complications of the vascular access site and new pacemaker implantation [8]. High-sensitivity cardiac Troponin T (hsTnT) as a biomarker for myocardial damage and N-terminal prohormone of brain natriuretic peptide (NT-proBNP) as a marker of heart failure were assessed pre- and post-interventionally. 

In a second analysis, we recorded the TR grade post-TAVR. Special attention was paid to patients with a switch in TR grade within seven days after TAVR: worseners of low-grade and improvers of high-grade TR. These groups were compared to their original group of low- or high-grade TR and the overall collective concerning short-, mid- and long-term-survival, VARC3-criteria and biomarkers. Furthermore, the risk factors for changes in TR grades were investigated.

### 2.4. Statistical Analysis

Continuous variables are presented as mean values with standard deviations and were compared between the groups using Wilcoxon rank-sum tests. Categorial variables are expressed as frequencies and were analyzed by the chi-squared test or Fisher’s exact test depending on the sample size. Survival time was illustrated using the Kaplan–Meier method and compared by the log-rank test. To assess the potential influence of all relevant variables upon survival, multivariable Cox regression analysis was performed at the two-year censored survival time. If patients met the absolute inclusion criteria, which was having a complete echocardiographic examination at baseline and discharge, missing values of up to 30% in the remaining variables were tolerated in the sense of pairwise case exclusion. All tests were two-sided at a 5% significance level. The statistical analysis was carried out using the software IBM SPSS Statistics (version 28) and R (version 3.6.2, package ‘survival’). 

## 3. Results

### 3.1. Survival after TAVR as a Function of Pre-Interventional TR 

The patients were divided into two groups according to their pre-interventional TR grade. Group 1 (low-grade TR, TR < 2) consisted of 684 patients, including 240 patients (31.0%) with non to trivial and 446 patients with mild TR (57.6%) at baseline. Patients with moderate (n = 76, 9.8%) and severe (n = 12, 1.2%) TR pre-TAVR were summarized in group 2 (high-grade TR, TR ≥ 2). Echocardiographic recordings were evaluated by three different cardiologists, with no significant difference in the raised grading (*p* = 0.275).

Baseline characteristics as a function of TR severity are shown in Table 1. Patients with high-grade TR were more likely to suffer from chronic atrial fibrillation (group 1: 40.2% vs. group 2: 77.5%; *p* = 0.001) and symptoms of heart failure with a significantly higher NYHA functional class (*p* = 0.003). They also featured a worse left and right ventricular function (*p* = 0.003 and *p* = 0.001), as well as a higher frequency of right atrial and right ventricular dilatation and pulmonary hypertension (*p* = 0.001 each). Overall, this resulted in a significantly higher pre-interventional risk evaluation (STS-Score 5.1% vs. 4.6%, *p* = 0.025, EuroScore II 6.5% vs. 4.7%, *p* = 0.001) for patients with high-grade TR compared to low-grade pre-interventional TR.

The most commonly implanted transcatheter aortic valves in our center were CoreValve Evolute R from Medtronic’s and SAPIEN-XT-3 from Edwards Lifesciences. Two-thirds of the procedures were performed by a transfemoral approach, followed by transaortic and transapical access.

**Figure 1:**
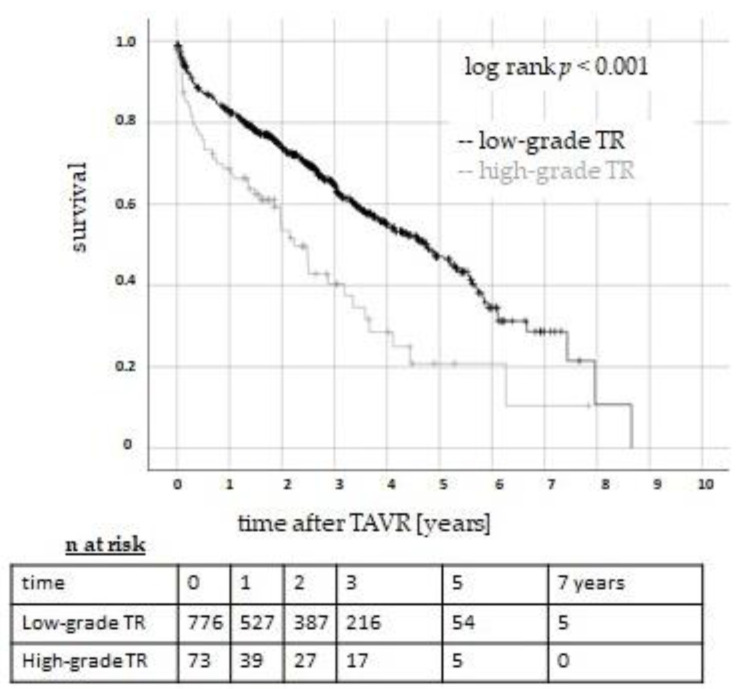
Survival after TAVR in patients with low- and high-grade TR. The Kaplan–Meier curves show a significant survival benefit in patients with pre-interventional low-grade (black line) TR compared to patients with high-grade (grey line) TR (log rank, *p* < 0.001). TR: tricuspid regurgitation. TAVR: transcatheter aortic valve replacement.

Mortality was defined as the primary endpoint. Patients with low-grade TR (group 1) tended to show a lower 30-day all-cause mortality (Table 2). Short- (two years) and long-term survival (up to eight years) was significantly higher in patients with low-grade pre-TAVR TR (*p* < 0.001; Figure 1). High-grade pre-interventional TR was therefore associated with impaired survival after TAVR in the analyzed collective.

TAVR-related complications in the two groups were assessed following VARC3 criteria (Table 2). Patients with low-grade TR had a significantly lower rate of postoperative bleeding (12.1% vs. 20.2%, *p* = 0.03). For the remaining VARC3 criteria, there was no significant difference between the groups. 

**Figure 2:**
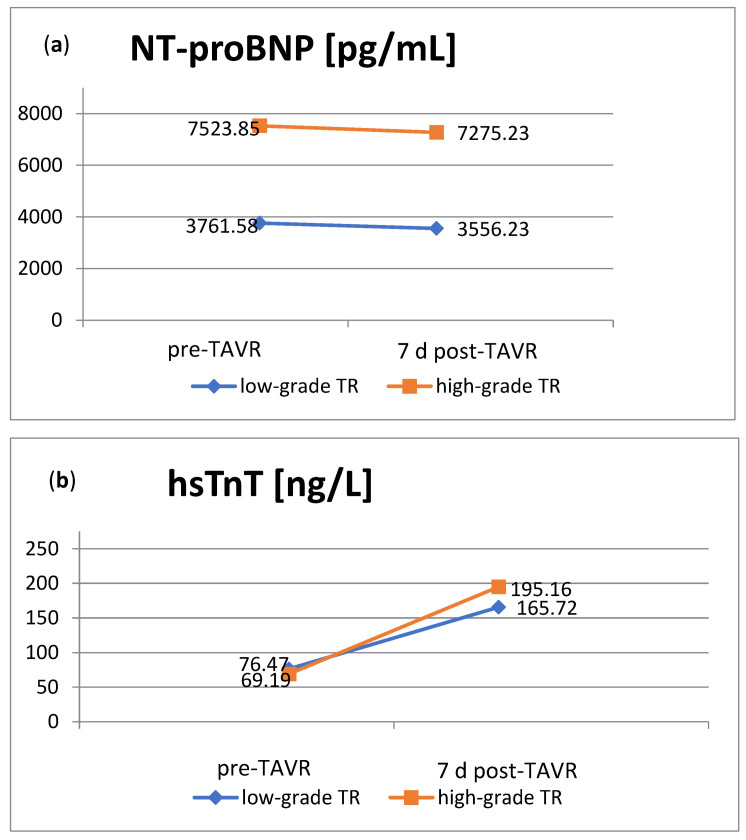
Heart-specific biomarkers pre- and post-TAVR in patients with low-grade or high-grade TR. (**a**). NT-proBNP concentrations were significantly higher in patients with high-grade TR both pre- and postoperatively (*p* < 0.001 each). (**b**). The HsTnT levels showed a postoperative increase, especially in patients with high-grade TR. NT-proBNP: N-terminal prohormone of brain natriuretic peptide; hsTnT: high-sensitivity cardiac Troponin T; TR: tricuspid regurgitation; TAVR: transcatheter aortic valve replacement.

The heart-specific biomarkers hsTnT and NT-proBNP were assessed pre- and seven days postoperatively. NT-proBNP was preoperatively elevated in both groups, but significantly higher in patients with high-grade TR (*p* = 0.001), which is in line with the NYHA functional class of heart failure. The postoperative level was comparable to the preoperative one, so the significant difference in NT-proBNP between the groups remained postoperatively (*p* < 0.001) (Figure 2a). The mean concentrations of hsTnT at baseline were comparable in patients with low- and high-grade TR. As expected, both groups showed a significant increase in hsTnT within seven days postoperatively, with a steeper rise in patients with high-grade TR (Figure 2b).

High-grade pre-interventional TR was associated with a worse outcome after TAVR. Independent of the grade of TR, several risk factors for a worse outcome after TAVR were identified in a multiple regression. These were age, atrial fibrillation, impaired renal function (measured by creatinine), arterial hypertension and chronic obstructive pulmonary disease (COPD) (Table 3). The previously mentioned heart-specific biomarkers hsTNT and NT-proBNP also turned out to be risk factors for mortality independent of the grade of TR. Right atrial and right ventricular dilatation, as well as impaired right ventricular function (TAPSE < 17 mm) and pulmonary hypertension (systolic PAP > 45 mmHg) were not independently associated with poor outcomes after TAVR. 

### 3.2. TR Changes after TAVR

Post-TAVR TR was assessed in an echocardiographic examination within seven days postoperatively. Patients were classified into four groups depending on whether their TR remained unchanged low- or high-grade, improved from a pre-interventional high-grade or worsened from a pre-interventional low-grade TR. 

**Figure 3:**
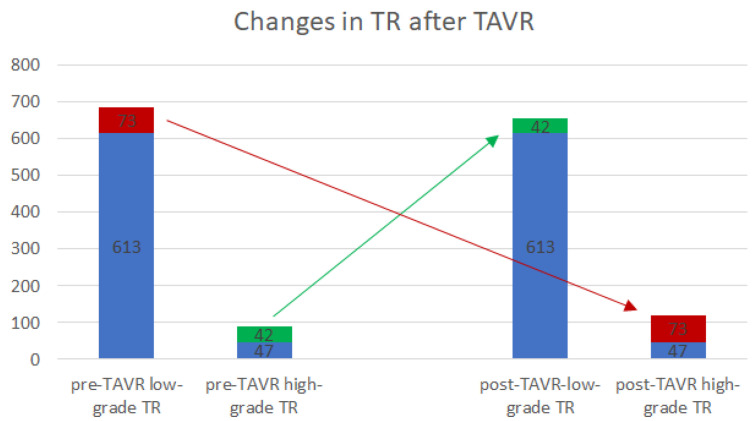
Changes in TR after TAVR. The diagram indicates the TR group affiliation pre-TAVR (left two columns) and 7 days post-TAVR (right two columns). Of 686 patients, 73 with low-grade pre-interventional TAVR deteriorated to a high-grade TR (red), whereas 42/89 patients with high-grade pre-operative TR improved to a low-grade level within seven days after TAVR (green). In 613/686 and 47/89 patients, the TR remained unaffected (blue). TR: tricuspid regurgitation; TAVR: transcatheter aortic valve replacement.

Changes in TR after TAVR are indicated in Figure 3. Out of 686 patients with a low-grade preoperative TR (TR < 2), 73 patients (10.6%) deteriorated to a high-grade TR within seven days after TAVR, so-called “worseners”. Meanwhile, in nearly half of the patients with a high-grade preoperative TR (n = 42, 47.2%), the regurgitation improved to a low-grade level (“improvers”) (Figure 3). 

**Figure 4:**
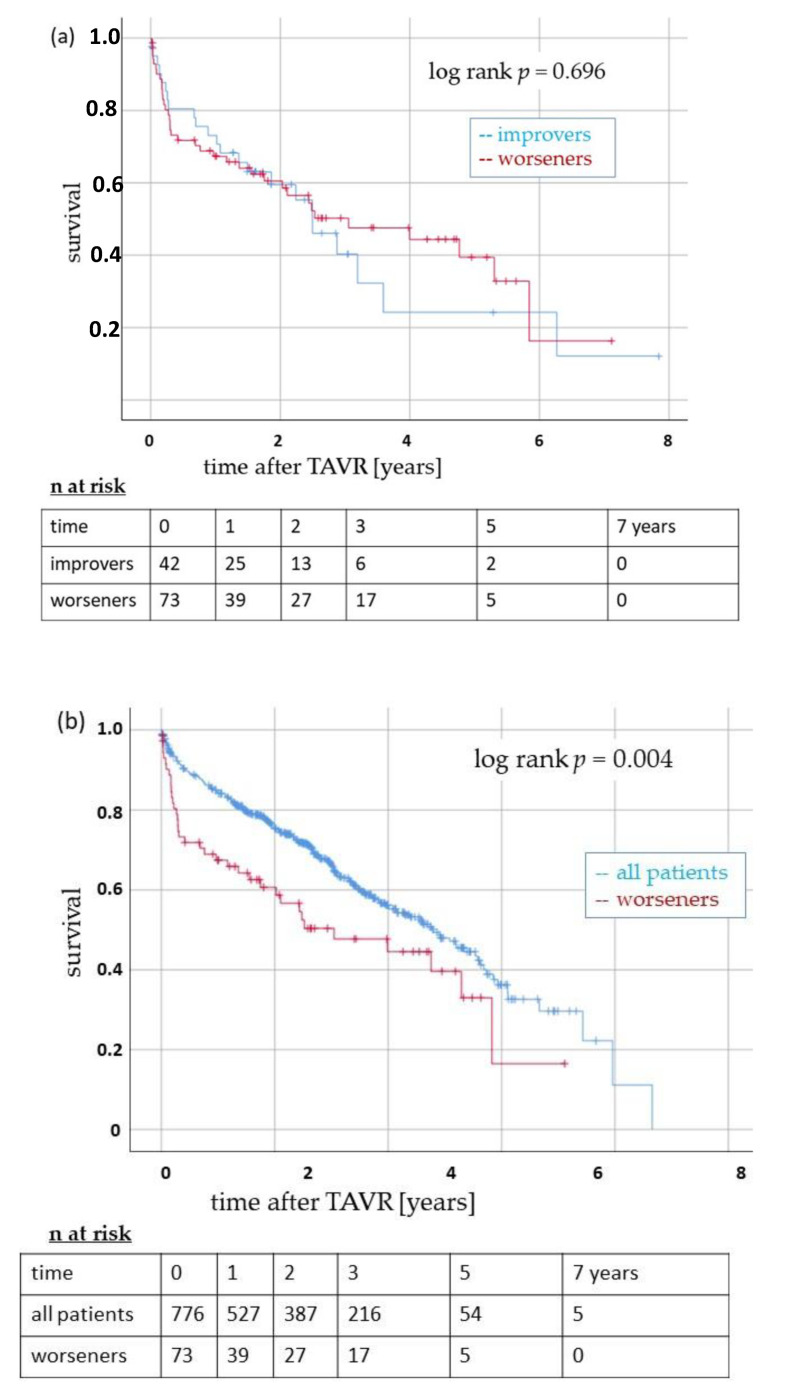
Survival after TAVR in patients with a post-interventional change in TR. (**a**): The Kaplan–Meier curves indicate long-term survival in patients with improved (blue line) or worsened (red line) TR within seven days after TAVR. There was no significant difference between the subgroups (log-rank *p* = 0.696). (**b**): Survival of patients with worsened TR after TAVR (red line) compared to all patients included in this study (blue line). Long-term survival was significantly impaired in the subgroup of worseners (log-rank *p* = 0.004). TR: tricuspid regurgitation. TAVR: transcatheter aortic valve replacement.

Survival according to post-TAVR TR was of primary concern. Although TR was improved in nearly half of the patients with pre-interventional high-grade TR, this did not result in a survival benefit in short- and long-term survival (Table 4, Figure 4). In contrast, more than 10 percent of patients with pre-interventional low-grade TR deteriorated. This resulted in a significantly lower short- and long-term survival in the subgroup of TR worseners compared to their original group of patients with pre-interventional low-grade TR and to the overall collective (Table 4, Figure 4).

Both subgroups did not differ from their original group or the overall collective concerning major postoperative complications according to the VARC3 criteria (Table 4).

Echocardiographic parameters and cardiac biomarkers in the subgroups of improvers and worseners were compared to their respective origin groups and the overall collective to define any potential markers for change in TR after TAVR. 

Compared to the overall collective and the group of patients with low-grade TR, right atrial and ventricular dilatation, as well as pulmonary hypertension and impaired right ventricular function, were significantly more frequent in the subgroup of TR worseners (Table 5). The subgroup of TR improvers did not show any significant difference in the mentioned echocardiographic criteria compared to patients with high-grade TR. Interestingly, even though differences between low-grade TR and high-grade TR were highly significant, there was no significant difference between the subgroup of improvers and the overall collective, with the exception of the reduced TAPSE (Table 5). 

**Figure 5:**
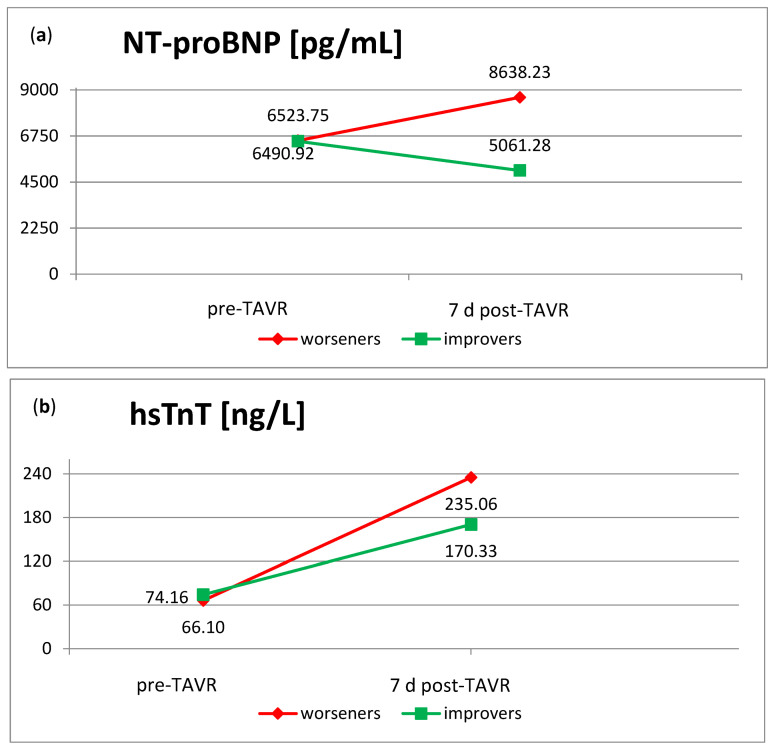
NT-proBNP and hsTnT in the subgroups of improvers and worseners. (**a**) NT-pro-BNP levels in the subgroup of worseners rose within seven days after TAVR. The group of improvers on the contrary showed a decrease. (**b**) The postoperative raise in hsTnT within seven days after TAVR was higher in the subgroup of worseners compared to the improvers. NT-proBNP: N-terminal prohormone of brain natriuretic peptide; hsTnT: high-sensitivity cardiac Troponin T; TR: tricuspid regurgitation; TAVR: transcatheter aortic valve replacement.

Future worseners already showed a higher level of NT-proBNP pre-interventionally compared to the group of patients with low-grade pre-interventional TR at baseline (6523.75 pg/mL vs. 3761.58 pg/mL) (Figure 2a and Figure 5a). In contrast to the low-grade TR group, where there was no significant change in BNP post-interventionally, NT-proBNP concentrations in the subgroup of worseners tended to rise within seven days after TAVR. The subgroup of improvers, on the contrary, already showed a lower level of NT-proBNP at baseline compared to their original group of patients with high-grade TR. They even presented a decrease in NT-proBNP within seven days after TAVR (Figure 2a and Figure 5a). Pre-interventional heart failure therefore seems to be associated with worsening TR after TAVR.

As shown in Section 3.1, hsTnT levels rose after TAVR. The subgroup of worseners thereby showed the highest increase, with more than three times the initial concentration (Figure 5b). Worsening of TR therefore seems to be in line with higher myocardial damage.

Within the group of worseners, multi-variable Cox regression was performed to define independent risk factors for worsening TR after TAVR. These were adipositas (measured by body mass index) and impaired right ventricular function (measured by TAPSE). Moreover, within the group of low-grade TR, which summarizes non to trivial and mild TR, patients were significantly more likely to suffer worsening of TR if mild TR was present pre-interventionally. Age, atrial fibrillation, impaired renal function, arterial hypertension and COPD were no risk factors for worsening of TR after TAVR in our analysis (Table 6). 

## 4. Discussion

The major findings of this comprehensive study were as follows: (1) Moderate to severe TR in pre-TAVR examinations is associated with an increased risk for mortality after TAVR on short-, mid- and long-term up to 8 years. (2) Patients with high-grade TR had a significantly higher morbidity as measured by common preoperative risk evaluation scores. (3) High-grade TR is also associated with a higher incidence of post-interventional bleeding. (4) Even though nearly half of the patients showed a reduction in TR severity after TAVR, this did not result in a survival benefit. (5) About 10% of patients had a progression of TR after TAVR, with a significant impact on survival. (6) Patients with progression of TR after TAVR had higher Nt-proBNP levels and a higher frequency of right atrial and ventricular dilatation and pulmonary hypertension at baseline compared to the overall collective. (7) Independent risk factors for worsening of TR after TAVR were adipositas, impaired right ventricular function and the presence of mild TR. 

In the context of treating aortic valve stenosis in elderly patients, multi-valvular disease is an important issue to deal with. In particular, the longtime forgotten tricuspid valve has attracted increasing interest as it may affect the outcome of aortic and mitral procedures. 

In an early study, Hutter and colleagues examined the presence of concomitant atrioventricular valve disease in 268 patients undergoing TAVR and described its negative impact on one-year survival [8]. This observation was confirmed by other groups and is consistent with our findings [4,5,9]. We were able to follow up this effect for long-term survival up to eight years, which is the longest follow-up to our knowledge. 

There are conflicting results on whether TR may be an independent predictor of mortality after TAVR [2]. In our multivariant analysis, independent risk factors for mortality after TAVR were age, atrial fibrillation, impaired renal function, hypertension and preoperative elevation of the biomarkers NT-proBNP and hsTnT, but not TR. Neither were right atrial and ventricular dilatations and impaired right ventricular function. The significantly higher morbidity group of patients with high-grade TR underlines the hypothesis raised by Khan et al. [1]. Al: TR itself might not be the reason, but it can be seen as a marker of underlying disease, leading to higher mortality after TAVR. 

Newly, we observed a significantly higher incidence of postoperative bleeding in patients with high-grade TR. This might be due to the higher rate of atrial fibrillation and the associated intake of anticoagulants, but also to higher co-morbidity. 

As concomitant TR is often a hemodynamic consequence of left-sided heart disease, TR improvements after TAVR can be imagined. We found that high-grade TR was reduced very fast within seven days after TAVR in nearly half of the patients with moderate to severe TR, confirming other studies [5,10,11,12]. Nevertheless, the improvement in TR did not result in a survival benefit compared to patients with unchanged high-grade TR in our 8-year follow-up. Wilbring et al. stated that the improvement of atrioventricular valve disease through TAVR did not have an impact on the symptoms of heart failure [10]. We can add to this observation that the levels of the marker of heart failure Nt-proBNP remained stable within seven days after TAVR in the overall collective. Nevertheless, we saw a decrease in NT-proBNP in patients with improved TR after TAVR and an increase in patients with worsening TR, reflecting the reduction respectively progression of heart failure going along. Further analysis of long-term development is necessary.

Yoshida et al. pointed out that atrial fibrillation and dilatation of the tricuspid annulus were predictors of persistent TR after TAVR [12]. In our analysis, we focused on patients experiencing not only persistent but also worsening TR after TAVR with its associated negative impact on short- and long-term survival. We were able to detect adipositas and impaired right ventricular function (TAPSE < 17mm) as independent risk factors for worsening of TR. Right atrial and right ventricular dilatation, as well as pulmonary hypertension, were also more frequent in the group of patients with worsening TR after TAVR. One could expect geometric alterations of the right ventricle to be in line with these findings, leading to heart failure after TAVR. Adequate heart failure therapy according to current guidelines is therefore crucial in these patients.

Together with the worsening of TR, a disproportionate postoperative increase in hsTnT was documented. In another analysis conducted at our center, we have already indicated that elevated hsTnT concentrations one year after TAVR were associated with a higher long-term mortality [13,14]. The postoperative hsTnT course could therefore be of interest for treating TR.

The right timing for treating TR in the context of TAVR remains unknown [2]. Current guidelines recommend concomitant tricuspid valve surgery for severe regurgitation if left-sided heart valve surgery is needed, but there are no such recommendations for valve interventions [1]. As we observed that nearly half of the patients with pre-TAVR high-grade TR improved to a low-grade level within seven days after TAVR, a combined intervention did not seem useful. On the other hand, impaired right ventricular function and high levels of NT-pro-BNP were associated with worsening TR after TAVR. We expect these patients to profit from cardiac recompensation therapy prior to aortic valve intervention.

### Limitations of the Study

Our study has several potential limitations: it is a large, single-center retrospective observational study with a primary focus on survival, but the choice of patients is prospective. This might result in a potential degree of selection bias with respect to the patient population. Nevertheless, all 775 patients had severe aortic valve stenosis and tricuspid regurgitation. Right ventricular function was assessed by TAPSE only; a more distinctive evaluation might have given even more insight into right ventricular physiology after TAVR. This also applies to the measured LVEF, which is more dependent on the afterload than newer methods to quantify left ventricular function, such as the left ventricular strain. Furthermore, we have chosen a more conservative grading of TR, as described in the former ESC/EACTS (2021) guidelines [1]. Choosing a suggested newer classification published by Hahn et al. (2022) might have impacted the results [15]. Due to technical improvements and increasing experience in the field of TAVR, the early start date of the study in January 2009 could have had a negative impact on the described results.

## 5. Conclusions and Impact on Daily Practice

Not only pre-interventional but also post-TAVR high-grade TR may be associated with a worse prognosis following TAVR. Resolving aortic valve stenosis can change concomitant TR, but an improvement does not appear to significantly impact short- and long-term survival. Worsening of TR after TAVR is possible and impairs the prognosis. Independent risk factors for worsening of TR after TAVR in our study were adipositas, impaired right ventricular function and the presence of mild TR. Moreover, pre-TAVR elevated cardiac biomarkers, as well as right atrial and ventricular dilatation and pulmonary hypertension, were associated with worsening of TR after TAVR. These patients might take advantage of optimal medicinal cardiac recompensational therapy prior to valve intervention. TR as well as cardiac biomarkers should be closely observed after TAVR to select patients for tricuspid intervention.

## Figures and Tables

**Table 1:** Baseline characteristics.

	Group 1 (Low-Grade TR) (n = 686)	Group 2 (High-Grade TR) (n = 89)	*p*-Value
Age (years)	81.3 ± 6.0	81.7 ± 6.4	0.431
Gender, male (%)	45.5 (n = 312)	38.2 (n = 34)	0.194
BMI, median (kg/m^2^)	26.0 (23.5–29.4)	25.7 (22.9–29.1)	0.268
Chronic-obstructive pulmonary-disease (%)	16.6 (n = 114)	16.9 (n = 15)	0.955
Diabetes (%)	32.1 (n = 220)	27.0 (n = 24)	0.330
Dyslipidemia (%)	53.2 (n = 365)	51.7 (n = 46)	0.787
Cerebrovascular disease (%)	19.9 (n = 136)	16.9 (n = 15)	0.506
chronic atrial fibrillation (%)	40.2 (n = 276)	77.5 (n = 69)	**0.001**
Previous surgery (%)	18.9 (n = 130)	14.6 (n = 13)	0.264
Hypertension (%)	91.0 (n = 624)	91.0 (n = 81)	0.988
pAVD (%)	17.8 (n = 122)	11.2 (n = 10)	0.122
NYHA functional class (%)	**(n = 681)**	**(n = 89)**	**0.003**
I	4.1 (n = 28)	1.1 (n = 1)	
II	24.9 (n = 171)	14.6 (n = 13)	
III	56.6 (n = 388)	62.9 (n = 56)	
IV	13.8 (n = 95)	21.3 (n = 19)	
**Risk-scores**	**(n = 686)**	**(n = 89)**	
Median STS-Score (%)	4.6 (2.9–7.0)	5.1 (3.8–7.9)	**0.025**
≤10%	89.5 (n = 613)	87.5 (n = 78)	
>10%	10.5 (n = 72)	12.5 (n = 11)	
Median EuroScore II	4.7 (3.0–7.8)	6.5 (4.0–9.7)	**0.001**
≤10.3%	84.2 (n = 577)	80.9 (n = 72)	
>10.3%	15.8 (n = 108)	19.1 (n = 17)	
**EF (%)**	**(n = 636)**	**(n = 87)**	**0.003**
<35%	8.6 (n = 59)	10.1 (n = 9)	
35–45%	13.3 (n = 91)	16.9 (n = 15)	
46–54%	17.2 (n = 118)	34.8 (n = 31)	
≥55%	53.6 (n = 368)	36.0 (n = 32)	
RA dilatation (%)	**(n = 628)** 20.1 (n = 130)	**(n = 85)** 48.2 (n = 41)	**0.001**
RV dilatation (%)	**(n = 627)** 14.5 (n = 91)	**(n = 85)** 41.2 (n = 35)	**0.001**
TAPSE < 17 mm (%)	**(n = 613)** 18.8 (n = 115)	**(n = 85)** 45.9 (n = 39)	**0.001**
PAPsys > 45 mmHg (%)	**(n = 570)** 26.5 (n = 151)	**(n = 80)** 53.8 (n = 43)	**0.001**
GFR (%)	**(n = 682)**	**(n = 89)**	0.313
<30 mL/min	12.1 (n = 83)	14.6 (n = 13)	
30–45 mL/min	21.6 (n = 148)	24.7 (n = 22)	
45–60 mL/min	35.4 (n = 243)	33.7 (n = 30)	
>60 mL/min	30.3 (n = 208)	27.0 (n = 24)	
	**(n = 684)**	**(n = 89)**	
Median Creatinine (mg/dL)	101.6 (79.7–130.1)	104.4 (82.2–133.5)	0.409
TAVR-access site (%)			0.877
Transfemoral (%)	67.2 (n = 460)	65.9 (n = 59)	
Transaortal (%)	20.0 (n = 137)	21.6 (n = 19)	
Transapical (%)	12.8 (n = 89)	12.5 (n = 11)	

(n in bold: total of analyzed patients). TR: Tricuspid regurgitation; TAVR: transcatheter aortic valve replacement; BMI: Body Mass Index; COPD, Chronic obstructive pulmonary disease; pAVD: Peripheral Artery Vessel Disease; NYHA: New York Heart Association, STS: Society of Thoracic Surgeons; Log ES I: Logistic Euroscore I; ES II, EuroScore II; EF: Ejektion-Fraction; RA: right atrium; RV: right ventricle; TAPSE: tricuspid annular plane systolic excursion; PAPsys: systolic pulmonary arterial pressure; GFR: Glomerular filtration rate. RA dilatation: area > 18 cm^2^, length > 45 mm RV dilatation: basal diameter > 42 mm, mid cavity diameter > 35 mm, longitudinal dimension > 86 mm.

**Table 2:** VARC3 criteria after TAVR in patients with low- and high-grade TR.

	Low-Grade TR (n = 686)	High-Grade TR (n = 89)	*p*-Value
30-day all-cause mortality (%)	19.1 (n = 131)	28.1 (n = 25)	**0.04**
Myocardial infarction (%)	1.2 (n = 8)	0 (n = 0)	0.31
Stroke (%)	3.1 (n = 21)	6.7 (n = 6)	0.49
Bleeding (%)	12.1 (n = 83)	20.2 (n = 18)	**0.03**
Acute kidney injury (%)	6.6 (n = 45)	16.9 (n = 5)	0.96
Complications vascular access site (%)	10.1 (n = 69)	11.2 (n = 10)	0.43
New pacemaker implantation (%)	7.2 (n = 50)	5.3 (n = 6)	0.53

VARC3: Valve Academic Research Consortium 3; TR: tricuspid regurgitation.

**Table 3:** Two-year survival according to risk factors.

Variable	Hazard Ratio	95% Confidence Interval	*p*-Value
Age (years)	1.058	1.022–1.096	0.002
Atrial fibrillation	1.787	1.210–2.641	0.004
Pre-interventional creatinine	1.003	1.000–1.005	0.002
Pre-interventional hsTNT	1.368	1.122–1.670	0.002
Arterial hypertension	3.245	1.190–8.846	0.021
COPD	2.189	1.391–3.446	0.001
Pre-interventional NT-proBNP	1.429	1.236–2.451	0.001

Non-significant: gender, right ventricular function, right atrial and right ventricular dilatation. NT-proBNP: N-terminal prohormone of brain natriuretic peptide; hsTNT: high-sensitivity cardiac Troponin T; TAVR: transcatheter aortic valve replacement; COPD: chronic obstructive pulmonary disease.

**Table 4:** VARC3 criteria in patients with worsened or improved TR within 7 days after TAVR.

	TR Worseners (n = 73)	*p*-Value (Worseners vs. Low-Grade TR n = 686)	*p*-Value (Worseners vs. All Patients n = 775)	TR Improvers (n = 42)	*p*-Value (Improvers vs. High-Grade TR n = 89)	*p*-Value (Improvers vs. All Patients n = 775)
30-day all-cause mortality (%)	31.5 (n = 23)	0.014	0.022	26.2 (n = 11)	0.591	0.191
Myocardial infarction (%)	1.4 (n = 1)	0.44	0.405	0 (n = 0)	0.500	0.998
Stroke (%)	1.4 (n = 1)	0.868	0.919	4.8 (n = 2)	0.680	0.351
Bleeding (%)	12.3 (n = 9)	0.477	0.569	11.9 (n = 5)	0.098	0.587
Acute kidney injury (%)	9.6 (n = 7)	0.198	0.189	0 (n = 0)	0.989	1.000
Complications vascular access site (%)	8.2 (n = 6)	0.705	0.720	7.1 (n = 3)	0.785	0.771
New pacemaker implantation (%)	2.7 (n = 2)	0.983	0.982	9.5 (n = 4)	0.680	0.310

*p*-values are given to compare the subgroups of worseners and improvers to their original group of low-grade or high-grade pre-interventional TR and to the overall collective. Thirty-day all-cause mortality is significantly higher in the subgroup of worseners. TR: tricuspid regurgitation; TAVR: transcatheter aortic valve replacement.

**Table 5:** Baseline echocardiography in patients with post-interventional change in TR.

	Worseners	All Patients	*p*-Value (Worseners Compared to All Patients)	Low-Grade TR	*p*-Value (Worseners Compared to Low-Grade TR)
RA dilatation (%)	55.1 (38/69)	23.9 (171/714)	0.001	20.1 (130/628)	0.001
RV dilatation (%)	42.0 (29/69)	17.7 (126/713)	0.001	14.5 (91/627)	0.001
PAPsys > 45 mmHg (%)	60.3 (41/68)	29.8 (194/651)	0.001	18.8 (115/613)	0.001
TAPSE < 17 mm (%)	47.6 (30/63)	22.0 (154/699)	0.001	26.5 (151/570)	0.001
	improvers	all patients	*p*-value (improvers compared to all patients)	high-grade TR	*p*-value (improvers compared to high-grade TR)
RA dilatation (%)	36.6 (15/41)	23.9 (171/714)	0.05	48.2 (41/85)	0.900
RV dilatation (%)	34.1 (14/41)	17.7 (126/713)	0.02	41.2 (35/85)	0.779
PAPsys > 45 mmHg (%)	33.3 (13/39)	29.8 (194/651)	0.324	45.9 (39/85)	0.911
TAPSE < 17 mm (%)	30.0 (12/40)	22.0 (154/699)	0.142	53.8 (43/80)	0.995

Future worseners featured a significantly higher frequence of right atrial and ventricular dilatation, pulmonary hypertension and impaired right ventricular function on pre-TAVR echocardiography, compared to the group of patients with low-grade TR at baseline and the overall collective. Future improvers did not show any significant difference from their original group of patients with high-grade TR or the overall collective. TR: tricuspid regurgitation. RA: right atrium. RV: right ventricle. PAPsys: systolic pulmonary arterial pressure. TAPSE: tricuspid annular plane systolic excursion. TAVR: transcatheter aortic valve replacement.

**Table 6:** Independent risk factors for worsening TR after TAVR.

Variable	Hazard Ratio	95% Confidence Interval	*p*-Value
Body mass index	1.128	1.041–1.223	0.003
TAPSE < 17 mm	4.371	2.4022–16.7988	0.006
Pre-interventional TR	1.330	1.041–1.622	0.004

Included variables: age, atrial fibrillation, creatinine, arterial hypertension, COPD, STS Score, EuroScore, gender, right atrial and right ventricular dilatation, pre-interventional hsTnT, pre-interventional NT-proBNP. TR = Tricuspid Regurgitation. TAPSE = Tricuspid annular plain systolic excursion. NT-proBNP: N-terminal prohormone of brain natriuretic peptide; hsTnT: high-sensitivity cardiac Troponin T, TAVR: transcatheter aortic valve replacement.

## Data Availability

The data presented in this study are available on request from the corresponding author. The data are not publicly available due to privacy.

## Abbreviations

AS	Aortic valve stenosis
ES	EuroScore
ESC	European Society of Cardiology
Hs-TNT	high-sensitivity troponin T
NT-proBNP	N-terminal pro-B-type natriuretic peptide
STS-Score	Society of Thoracic Surgeons Score
TAVR	Transcatheter aortic valve replacement
TR	Tricuspid Regurgitation
